# The Present Status of the Rubber Question

**Published:** 1869-02

**Authors:** 


### Editorial.

### THE PRESENT STATUS OF THE RUBBER QUESTION.

Inquiries are constantly being made in reference to this subject, and to those most interested to know, we have, from time to time, by circulars and letters, endeavored to convey the desired information, so far as it was possible to do so. A great many questions have been asked, even by those entitled to full information, that it was impossible for us to answer, and, in addition to this, a great deal of information called for, and even demanded, by those to whom the Executive Committee were under no obligations; but even in such cases as much attention has been given as it was possible to bestow.

We have been censured for not keeping a constant dribble of this matter through the pages of the REGISTER. All we have to say upon this point is, that it is always a matter of regret when circumstances require its introduction.

As all the readers of the REGISTER are aware, the suits that were pending against Dentists, in this city and Chicago, for infringement of the Goodyear patent in the use of vulcanizable rubber for Dental purposes, have been decided, in every point, adversely to the defendants, and all who have read Judge Leavitt's decision know upon what grounds. The Rubber Company, within the last twelve months, have very much modified their demands and requirements. The most objectionable features have been taken away altogether, viz: the keeping and rendering to the Company a detailed account of all work done, embracing the names and residence of patients. They have also remitted very much of their claims for settlement and license fee, making the latter a definite thing, instead of an extravagant royalty upon all work done.

The duties of the committee having this matter in charge for the last two years and a half have been very arduous, consuming a large amount of valuable time that might have been better employed in some other direction, as well as dividing and diverting attention and effort that should have been employed for other purposes. In view of all these things, the Committee, after the most mature deliberations together, and with their able counsel, unanimously agreed to abandon further litigation, and to close the whole matter as early as possible, by the payment of costs and expenses, and the return of all notes in our possession to their respective makers. In order to meet the costs fully, a call for a payment of dues upon delinquent subscribers was necessary.

The question is frequently asked: Why was not one of these suits carried to the Supreme Court? We think that question already fully answered, but we will say further, that such an appeal would not prevent injunctions, issuing from the Circuit Courts, which would be equivalent to the collection of damages and the exaction of license fees, neither of which could be regained, whatever might be the final result.

Under all these circumstances, the Committee could not think for one moment of entering into a three or four additional years' of vexatious, time destroying litigation, merely to sustain that which, in some respects, is a most miserable humbug. We could not wish our worst enemy as sore a wound as our profession received by the introduction of rubber for artificial dentures.

